# A Stakeholder-Engaged Approach to Development of an Animal-Assisted Intervention for Obesity Prevention Among Youth With Autism Spectrum Disorder and Their Pet Dogs

**DOI:** 10.3389/fvets.2021.735432

**Published:** 2021-11-11

**Authors:** Deborah E. Linder, Sara C. Folta, Aviva Must, Christina M. Mulé, Sean B. Cash, Eli D. Halbreich, Candice Colón, Sandy Sullivan, Edward Sanabria, Debra Gibbs, Terri Farrell

**Affiliations:** ^1^Tufts Institute for Human-Animal Interaction, Cummings School of Veterinary Medicine at Tufts University, North Grafton, MA, United States; ^2^Friedman School of Nutrition Science and Policy, Tufts University, Boston, MA, United States; ^3^Department of Public Health & Community Medicine, Tufts University School of Medicine, Boston, MA, United States; ^4^Division of Developmental Behavioral Pediatrics, Department of Pediatrics, University of Rochester Medical Center, Rochester, NY, United States; ^5^Division of Developmental Behavioral Pediatrics, Department of Pediatrics, Tufts University School of Medicine, Boston, MA, United States; ^6^Department of Psychology, Tufts University School of Arts and Sciences, Medford, MA, United States; ^7^LEARN Behavioral, Baltimore, MD, United States; ^8^Massachusetts Bay Transportation Authority, Boston, MA, United States; ^9^Perfect Piece LLC, Marlborough, MA, United States; ^10^Cummings School of Veterinary Medicine at Tufts University, North Grafton, MA, United States; ^11^The Shriver Center, The University of Massachusetts Chan Medical School, Worcester, MA, United States

**Keywords:** human-animal interaction, stakeholder engagement, patient engagement, Autism Spectrum Disorder, obesity, animal-assisted intervention, intervention program methods and outcomes, applied behavior analysis

## Abstract

Stakeholder involvement in research has been demonstrated to increase the effectiveness, validity, and quality of a study. This paper describes the engagement of a stakeholder panel in the development and implementation of an animal-assisted intervention (AAI) assessment and program for children diagnosed with Autism Spectrum Disorder (ASD). Canines for Autism Activity and Nutrition (CAAN) aims to promote physical activity and wellness among children diagnosed with ASD by integrating activities with their pet dog during the child's ongoing Applied Behavioral Analysis (ABA) in-home therapy sessions. Feedback from stakeholders guided program development at each stage of the research process, including this publication. Utilizing a stakeholder-informed approach was essential for the development of assessment tools, program materials, and program design. Methods that may assist others to effectively partner with stakeholders to implement an AAI among children diagnosed with ASD or related disorders are described.

## Introduction

Stakeholder engagement is a rapidly expanding approach to program development and applied research which has been demonstrated to increase the effectiveness, validity, and quality of a study (1, 2) and has been embraced by the public health community (3, 4). Advocates for a stakeholder-engaged approach assert that the approach can improve the scope and quality of research questions, design, and results (5, 6). However, one criticism of a stakeholder-engaged approach—especially in studies that involve a large numbers of stakeholders—is the potential difficulty in cross-study applicability due to a lack of quantitative measures of stakeholder engagement (7). While this study intends to evaluate the engagement of stakeholders qualitatively, there have been advances in the development of validated quantitative stakeholder engagement measures [e.g., (8)]. In human-animal interactions, and more specifically, animal-assisted interventions (AAI), stakeholder engagement can be particularly impactful due to the multidisciplinary nature of the field. Engagement from a diverse group of stakeholders for AAI has the potential to improve the health and well-being of all participants including the children, family members and caregivers, healthcare providers, and the animals that are being partnered with for the intervention.

The purpose of the AAI, Canines for Autism Activity and Nutrition (CAAN), is to promote physical activity and healthy eating behaviors in youth with Autism Spectrum Disorder (ASD) delivered by applied behavioral analysis (ABA) therapists with the family pet dog in home settings. Children and adolescents diagnosed with ASD are at an increased risk of overweight or obesity compared to their peers (9, 10). Food selectivity for children diagnosed with ASD can result in poor quality diets (11, 12), which increases the risk for obesity over time (13). Furthermore, physical activity levels for children with ASD may be lower than their neurotypical peers for a variety of reasons, including difficulties with motor control (14) and difficulties with social interactions (15, 16). Many of these characteristics that contribute to obesity also render traditional weight loss programs for neurotypical children unsuitable for most children with ASD. Given the unique needs of this population, diverse stakeholder engagement was deemed critical to develop appropriate and feasible strategies to engage child and dog dyads. The program was designed to facilitate physical activity, social connection, mutual enjoyment, and encourage children to make healthier food choices by teaching them proper nutrition for their dog through rules and goals (e.g., portion control) that children can also apply to their eating habits.

In child obesity prevention programs, stakeholder engagement in the development phase has been shown to support positive outcomes and improve intervention acceptance, inclusion of underrepresented populations, and participant engagement (17, 18). When applying obesity prevention to the population of children with ASD and employing the novel methodology of AAI, development without the engagement of diverse stakeholders may be ineffective, or even counterproductive, due to the complex and multidisciplinary nature of the considerations outlined above.

The purpose of this paper is to describe the use and impact of a stakeholder panel in the development and planned implementation of the CAAN obesity prevention program for youth with ASD and their pet dogs. Specifically, we hope to provide information that will be helpful to other researchers considering a stakeholder-engaged approach to their investigations and to lay the groundwork for future research. The paper will explore how a stakeholder panel can be used effectively, discuss lessons learned during the process, and serve as additional support for the benefits of stakeholder engagement, particularly in AAI.

## Methods

### Stakeholder Recruitment and Initial Engagement

At the beginning of the CAAN project, co-investigators identified that stakeholders would be critical to the successful development of the intervention. At the time of proposal development for grant funding, stakeholder engagement was included as a prominent aspect of the research approach, incorporated into the aims and budget of the grant. The individuals identified with relevant experience and expertise in one or more aspects of the project were invited to serve on the stakeholder panel, provide feedback on the proposal, and provide letters of support to submit with the proposal. In addition, two ABA service providers were contacted to serve as recruitment sites in the active pilot phase of the project. Following this partnership, an additional stakeholder from one of the agencies was identified and asked to join the stakeholder panel after the grant was funded and intervention development began.

### Investigative Team

The investigative team includes a diverse group of multidisciplinary faculty members that identified stakeholders who would complement the investigative team's strengths and backgrounds. Dr. Linder is a board-certified veterinary nutritionist at the Tufts University Cummings School of Veterinary Medicine as well as co-director of the Tufts Institute for Human-Animal Interaction who brought knowledge and experience in AAI, pet obesity, and nutritional management to the project. Dr. Mulé is a pediatric psychologist who at the time of the study provided care at the Center for Children with Special Needs at Tufts Medical Center. She also has extensive expertise in the diagnosis and treatment of ASD, physical activity promotion in autism, as well as stakeholder engagement. Dr. Must is a nutritional epidemiologist whose research efforts focus on observational and intervention studies of obesity in vulnerable populations, with an emphasis on youth with developmental disabilities. She serves as co-director of the Department of Health and Human Services Maternal and Child Health Bureau's Healthy Weight Research Network for Children with Autism and other Developmental Disabilities. She brought expertise on interventions for this population. Dr. Folta is a behavioral scientist who developed and disseminated the Strong Women–Healthy Hearts Program, a community nutrition and physical activity program for older women. She brought expertise in qualitative research methods and has extensive experience conducting in-depth interviews among youth with autism. Dr. Cash is an economist whose work with interdisciplinary teams focuses on economic aspects of behavior around food and nutrition and is the parent of a child with autism. His work includes investigating how children behave when engaging in autonomous food purchasing activities and developing child food literacy measures. Drs. Mulé, Must, and Folta all have previously used stakeholder-engaged approaches in their research.

### Composition of the Stakeholder Panel

Based on the research and clinical background of the investigative team, an objective for the stakeholder panel was to include perspectives from individuals with practical and personal experience with various areas of the project: specifically, ASD, ABA, and AAI. To this end, the diverse stakeholder panel included: a provider of AAI (Debra Gibbs), two Board Certified Behavior Analysts (BCBAs; Dr. Candice Colón and Edward Sanabria), two parents of children with ASD (Sandy Sullivan and Terri Farrell, who also is project director of the Autism Insurance Resource Center at the University of Massachusetts Chan Medical School), and a young adult with ASD (Jeremy Nisbet, along with his mother, Jennifer Nisbet). Debra Gibbs, Animal-Assisted Programs Coordinator at the Tufts Institute for Human-Animal Interaction, is a licensed Pet Partners Handler instructor with years of experience in evaluating the safety and efficacy of human-animal partnerships for AAI. More specifically, she is also involved in AAI work for children with disabilities. Dr. Candice Colón is a BCBA-D who specializes in ABA research and the assessment and treatment of behavioral challenges for children diagnosed with ASD and provided a hands-on perspective to the panel. Edward Sanabria, also a BCBA, specializes in the integration of behavior analytic interventions in the home and community settings for children diagnosed with ASD. The two parents of children with ASD are Sandy Sullivan and Terri Farrell, each with different backgrounds and insight into raising a child with ASD. Sandy and her daughter primarily experienced challenges with language development and behavior regulation while issues with weight maintenance were emerging in the background. Terri Farrell works for the Autism Insurance Resource Center at the University of Massachusetts Chan Medical School and is the parent of a young adult with ASD and owner of two pet dogs. She has been active in several autism advocacy groups, has personal experience observing the relationships between pet dogs and her child with autism, and has professional experience working with other families and insurance agencies. Our final stakeholders were, Jeremy Nesbit, a young adult with ASD, who brought his valuable perspective on the intervention and emphasized key areas of social validity along with his mother, Jennifer Nesbit, who attended meetings with him.

### Engagement and Communication Plan

The stakeholder panel met with the investigative team in-person or by telephone or videoconference four times over the project year (with email correspondence between meetings to review and revise materials). Agendas for each meeting were created to follow the project's larger goal for intervention development (Table 1).

**Table 1:** Stages of CAAN Development.

**Stage number**	**Description**
1.	Incorporation of relevant literature on physical activity promotion, nutrition, AAI, and education for children with ASD
2.	Development of a facilitator's guide draft for ABA therapists
3.	Development of a draft AAI, including three activity modules
4.	Development of a post-intervention interview guide and usability scales
5.	Content review by our stakeholder panel
6.	Refinement of the draft materials based on stakeholder feedback in preparation for pilot testing

## Results

Stakeholder engagement impacted CAAN in three major domains: development of materials, development of program modules and planned implementation of the intervention, and planned assessment of the intervention.

### Development of Materials

#### Facilitator's Guide

The first stakeholder meeting was used to review the facilitator's guide. A draft was shared with stakeholders in advance of the meeting so that members had time to prepare thoughtful feedback. During the meeting, stakeholders were asked to share their initial impressions of the facilitator's guide, feasibility concerns/appropriateness of the intervention for children with ASD, and personal experiences related to working with children with ASD and AAI. A discussion followed for how these experiences should be incorporated or accounted for in the guide. Following this meeting, the facilitator's guide was revised based on stakeholder input. Specifically, feedback from stakeholders informed who would be the primary implementers of the program and eligibility considerations for participating families and ABA therapists. The feedback led to the refinement of the specific skills and qualities to look for in an ABA therapist, and to focus more heavily on the ABA therapist. Additionally, the stakeholders provided input on the usability of the materials, such as including a table of contents, which was added as the result of a stakeholder suggestion.

The third stakeholder meeting was used to review and refine the final draft of materials. The revised facilitator's guide was shared with the stakeholder panel prior to meeting. During the meeting, stakeholders were asked to share their perspectives on how the guide could be further strengthened. At this meeting, barriers and facilitators to the success of the program were discussed and identified, which led to the addition and improvement of an additional section in the guide for strategies to troubleshoot challenges that could arise, such as the dog as a potential distraction during treatment and on-site injuries that would include financial and liability considerations. Critical factors of support that stakeholders identified were clarity and ease of use of the guide and that the treatment be evidence-based. Following this meeting the guide was revised based on stakeholder input. Specifically, the guide was revised to include specific information for BCBAs, which clarified how the program could be integrated by the practitioner into their client's existing treatment plan. See Table 2 for a description of the specific benefits and impact of stakeholder engagement on the development of materials.

**Table 2:** Specific Benefits and Impact of Stakeholder Engagement on the CAAN Program.

**Materials development**	**Program implementation**	**Program assessment**
- Expanded safety considerations for both the client and the pet - Increased clarity and specificity based on personal experiences and preferences from individuals with ASD - Addition of a section specifically for BCBAs in facilitator's guide - Addition of troubleshooting section for anticipated challenges including liability concerns	- Refinement of inclusion/exclusion criteria - Refinement of orientation procedures for the family, client, and canine - Modification to ABA therapist training around canine stress signals - Added protocol specificity and flexibility - Enhanced application of study activities to life skills - Revised appropriate behavioral reinforcers - Refinement of recommendations for locations and equipment	- Simplified and clarified interview guide questions - Enhanced focus of the interview questions - Inclusion of outcomes of importance for future program revision

### Development and Planned Implementation of CAAN

#### Program Modules

The second stakeholder meeting was used to review the three modules that comprise the AAI. During this meeting, stakeholders were asked to provide their feedback that focused on the appropriateness of the intervention for children with ASD in an ABA setting as well as any feasibility concerns. Following this meeting, modules were revised based on stakeholder input. Specifically, modifications were made to make the module instructions more specific, as a stakeholder noted that many children diagnosed with ASD generally prefer for everything to be as concrete as possible in a plan. Additionally, based on feedback, contingency plans were included for what would happen if implementation of the module did not proceed as described. For example, if the task is too difficult for the child or if the dog has eaten just prior to the session. Once revisions were made, revised documents were circulated to the stakeholder panel for a second round of review.

#### Implementation Plan

During the third meeting, the modules and implementation plan were also reviewed with stakeholders to further strengthen this component. As with the facilitator's guide, the critical factors that were focused on—to increase the likelihood of support and commitment from ABA service providers—were clarity, minimal response effort, and ease of integration into existing ABA treatment plans. The modules were again revised after this meeting to incorporate this stakeholder input. In particular, the modules were amended to allow BCBAs the flexibility to individualize ABA teaching strategies used to implement the AAI program.

#### Modification of Implementation Plan for COVID-19

Lastly, a fourth meeting was held to discuss how the program could be adapted in relation to the COVID-19 pandemic. This meeting was conducted via videoconference. The transition to videoconference could potentially increase the feasibility of organizing stakeholder meetings, especially for stakeholders who live far away. Stakeholders and the investigative team acknowledged and discussed the specific difficulties that COVID-19 could bring to families with a child with ASD and further modified the program modules to incorporate mask-wearing and social distancing and to ensure compliance with Centers for Disease Control and Prevention (CDC) guidelines for the safety of all participants during in-home ABA therapy. The decision was made to rely on the ABA service providers to determine when families felt comfortable having in-person sessions again, even after any federal or state social distancing measures or guidelines were eased. Additionally, the decision not to transition the program to a virtual format was discussed with stakeholders; it was agreed upon that even if the research could be conducted in person, the results could be influenced by the disruptive nature of COVID-19. See Table 2 for a description of the specific benefits and impact of stakeholder engagement on program implementation.

### Planned Assessment of CAAN

#### Interview Guides

During the fourth meeting, stakeholders were also asked to share their opinions on the post-intervention in-depth interview guide and usability scales. The usability rating scales, namely the Usage Rating Profile (19) and Child Usage Rating Profile (20)—modified slightly for this program—are brief scales designed to ascertain the usability and acceptability of interventions for adults and children, respectively. The stakeholders were asked if there were any additional areas they would like included in the interview guides. Following this meeting, interview guides were revised based on stakeholder input. For example, the questions for the children with ASD were made more specific, and more questions were added that focused on relationships with the pet dog. These changes would not have been considered in the absence of stakeholder feedback, and the qualitative data collected from interviews would have been less focused. See Table 2 for a description of the specific benefits and impact of stakeholder engagement on program assessment.

### Feasibility and Impact of Stakeholder Engagement

A stakeholder engagement approach was essential for the development of the CAAN program; it also proved to be very feasible, as the benefits to program design, implementation, and distribution far outweighed the monetary or time costs of engaging the stakeholder panel. As detailed in Table 2, the stakeholder panel brought their combined experience to help ensure the program was designed, implemented, and distributed in the most efficient and effective manner possible.

## Discussion

Stakeholder input was integral to the development of planned assessment and intervention inclusive of program materials to ensure applicability and feasibility of the CAAN program. The facilitator's guide, program modules, and interview guide were greatly strengthened and improved by feedback and guidance from the stakeholder panel. Collaboration with a diverse stakeholder panel and allowing each member of the panel to freely contribute new insights (that might have otherwise been overlooked by an investigative team) were invaluable, especially when developing novel interventions and pilot studies. The importance of the multidisciplinary aspect of the stakeholder panel cannot be overstated, as members of the panel were able to draw upon their unique expertise to provide input and feedback. Various technical aspects of the intervention were modified and further developed to have the greatest feasibility for use with the respective population within their current individualized treatment plan. In addition to technical feedback, the stakeholder panel was instrumental in guiding the larger study design and had a major role in developing the intervention. The experiences described in this paper highlight the impact and importance of stakeholder engagement in complex interventions such as AAI in populations with unique needs such as children with ASD. It is the intention of the authors that publication of this stakeholder-engaged approach will contribute to the growing body of evidence in support of stakeholder inclusion in the design and implementation of future research.

### Future Directions

The next step in the CAAN program will be to pilot the CAAN intervention among families that have a child with ASD, a family pet dog, and currently receive in-home ABA therapy. As part of that pilot implementation, an assessment will be conducted with ABA service providers, parents, and youth participants with ASD utilizing the stakeholder-informed interview guides. Future plans for the stakeholder panel include inviting stakeholders to participate in the next stage of the CAAN research program. This will include reviewing findings from the assessment of the pilot intervention and consideration of additional grant proposals and study design for a future randomized clinical trial of the intervention. Lastly, stakeholders will also have the opportunity to provide input on future dissemination of results. For publications such as this one describing stakeholder engagement, stakeholders have been engaged as co-authors. The project stakeholders also intend to participate in disseminating results to non-scientific audiences such as parent support groups and advocacy groups. They may also present results at local or national meetings of ASD, ABA or AAI professional organizations.

Future directions also include an evaluation of the impact of the stakeholder panel on the effectiveness of the program and its outcomes, which could be achieved by including members of the research team and members of the stakeholder panel in qualitative interviews as a part of the planned future pilot intervention. In addition to the interviews, the research team will assess the degree to which study materials and procedures changed based on advice from the stakeholder panel. Additionally, engagement with study findings will be considered, including the relative reach of traditional dissemination vs. the more holistic approach taken by the stakeholder panel. Because the research team has involved the stakeholder panel at all stages of the study, it will be difficult to uncouple the effectiveness of the program from the panel. However, these measures should provide valuable insights into the effectiveness of a stakeholder-engaged approach. Our intent is to pilot the intervention itself with families and to evaluate the overall stakeholder-engaged approach and its impact on the program outcomes.

## Conclusions

Utilizing a stakeholder-informed approach to develop an AAI increased the feasibility and potential effectiveness of the intervention through input into three main activities of the research team: materials development, program implementation, and development of assessment tools. The research team believes that this stakeholder-engaged approach will lead to greater potential uptake by ABA agencies, which will make the intervention more accessible to children receiving ABA therapy for treatment related to their ASD diagnosis. The experiences outlined in this paper highlight the impact and importance of stakeholder engagement in complex interventions such as AAI in populations with unique needs such as children diagnosed with ASD. The authors of this paper, which include the investigative team and members of the stakeholder panel, would like to emphasize a call to action for future AAI to consider incorporating a stakeholder panel into their study designs to drive the social validity and feasibility of such interventions with various unique populations.

## Data Availability Statement

The original contributions presented in the study are included in the article/supplementary material, further inquiries can be directed to the corresponding author.

## Author Contributions

DL, SF, AM, CM, and SC designed research. DL, SF, AM, CM, SC, EH, CC, SS, ES, DG, and TF contributed to manuscript preparation. DL had primary responsibility for final content. All authors read and approved the final manuscript.

## Funding

The project described was supported by the Tufts Institute for Global Obesity Research (TIGOR), the National Center for Advancing Translational Sciences, National Institutes of Health, Award Number UL1TR002544, and the Healthy Weight Research Network, Health Resources and Services Administration, and Maternal and Child Health Bureau (UA3MC25735).

## Author Disclaimer

The content is solely the responsibility of the authors and does not necessarily represent the official views of the NIH.

## Conflict of Interest

DL is a faculty advisor and DG is the program coordinator for a Pet Partner Community Partners group at Tufts University. DG is also a Pet Partners trainer and evaluator. CC was employed by company Behavioral Concepts, Inc. ES was employed by company Perfect Piece LLC. The remaining authors declare that the research was conducted in the absence of any commercial or financial relationships that could be construed as a potential conflict of interest. The funders were not involved in the study design, collection, analysis, interpretation of data, the writing of this article or the decision to submit it for publication.

## Publisher's Note

All claims expressed in this article are solely those of the authors and do not necessarily represent those of their affiliated organizations, or those of the publisher, the editors and the reviewers. Any product that may be evaluated in this article, or claim that may be made by its manufacturer, is not guaranteed or endorsed by the publisher.
